# Deficiency disrupts photoreceptor viability and synaptic integrity in a choroideremia mouse model

**DOI:** 10.1038/s41419-025-08336-y

**Published:** 2025-12-22

**Authors:** Jiangbo Yan, Tianlu Zhang, Yuxin Du, Zeyuan Pu, Yazhen Ma, Yang Wu, Yin Shen

**Affiliations:** 1 Department of Ophthalmology, Tongji Hospital, Tongji Medical College, Huazhong University of Science and Technology, Wuhan, 430030 China; 2 Eye Center, Renmin Hospital of Wuhan University, Wuhan University, Wuhan, Hubei China; 3 State Key Laboratory of Magnetic Resonance Spectroscopy and Imaging, Wuhan Center for Magnetic Resonance, Innovation Academy for Precision Measurement Science and Technology, Chinese Academy of Sciences, Wuhan, 430071 China; 4 Frontier Science Center for Immunology and Metabolism, Medical Research Institute, Wuhan University, Wuhan, Hubei China

**Keywords:** Cell death in the nervous system, Retina

## Abstract

Choroideremia, an X-linked retinal dystrophy causing progressive vision loss, arises from deficient Rab escort protein-1 (REP1), critical for the prenylation of Rab proteins. The precise mechanisms linking REP1 dysfunction to retinal degeneration remain unclear. Here, we generated conditional REP1 knockout mouse to model choroideremia and dissect REP1’s role in retinal homeostasis. Histological analysis revealed severe photoreceptor (PR) layer thinning by postnatal day 30, accompanied by disrupted synaptic architecture in the outer plexiform layer. Electroretinography revealed significant visual dysfunction, characterized by substantially reduced scotopic a-wave and b-wave amplitudes, indicating PR and perhaps bipolar cell (BC) impairment. RNA-Sequencing and immunofluorescence labeling showed downregulation of PR, synaptic, and phototransduction-related molecules as well as disrupted structural integrity of PRs. Transmission electron microscopy revealed ultrastructural synaptic defects, including shortened synaptic ribbons and loss of invaginated triads. Our findings establish an essential role for REP1 in maintaining PR viability and synaptic connectivity. Furthermore, we demonstrated that retinal degeneration in CHM mouse is associated with the activation of microglia mediated by the NF-κB pathway, suggesting that targeting neuroimmune is one of the potential therapies for CHM patients.

## Introduction

Choroideremia is an X-linked, recessive inherited retinal disease, estimated to affect approximately 1 in 50,000 males. The condition is characterized by progressive atrophy of the retinal pigment epithelium (RPE), choroid, and photoreceptors (PRs) [1]. Currently, there are no effective treatments. While traditional models suggest that RPE degeneration is the initiating event [1, 2], recent adaptive optics imaging studies demonstrate that PR outer segment (OS) abnormalities occur as early as the first decade in patients with preserved central vision [3], challenging classical models of the pathogenic sequence. In patients with choroideremia, vision loss is progressive and inevitably leads to blindness. Typically, children affected by the disease report difficulty seeing at night in their first or second decade of life, and in their 20 s become aware of the loss of peripheral vision [1, 4].

Mutations in the *Chm* gene, which encodes Rab escort protein 1 (REP1), result in choroideremia [5]. REP1 is a crucial protein involved in the prenylation and intracellular trafficking of Rab GTPases, playing a vital role in vesicular transport in both the retina and RPE [6, 7]. Structural analyses reveal that common REP1 mutations induce misfolding of the Rab-binding domain, impairing both enzymatic and chaperone functions, a mechanism distinct from simple loss of prenylation activity [8]. This explains why some missense variants show residual REP1 activity despite causing severe phenotypes. Sequence variations, translocations, point mutations, minor deletions, insertions, nonsense mutations, and frameshift mutations are some of the variants in *Chm* that cause choroideremia [5]. Although most choroideremia-causing mutations are thought to be functionally null, there can still be genetic heterogeneity within a single family. The extent of *Chm* mutations and the resulting disease severity are not clearly correlated [9].

The sequence of pathogenic events in choroideremia is controversial. One hypothesis is that the disease initially manifests as RPE degeneration, perhaps with independent and concurrent PR outer segment (OS) degeneration, and later choroidal atrophy [2, 10]. However, there is also evidence of severe abnormalities at the PR level, particularly the OS, that clinically precede overt, localized RPE abnormalities [11]. The pathogenesis of *Chm* is complex and unidentified, as three interdependent eye layers (choroid, PRs, and RPE) are affected. One idea is that initiation of *Chm* starts in one layer, leading to a cascade effect on the other layers. In one study [12], CHM carrier (*Chm*^null/WT^) mouse were crossed with six3-Cre and inducible MerCreMer transgenic lines, resulting in *Chm* knockout specific to neural retina, or RPE, respectively. They demonstrated that distinct subsets of Rabs are underprenylated in the RPE and the neuroretina, suggesting that degeneration of PRs and the RPE are independent processes caused by the loss of REP1 function. However, PR functional deficits and cell death occur much earlier when RPE degeneration is also present, suggesting that the diseased RPE accelerates PR degeneration [13].

In humans with CHM/REP1 loss-of-function mutations, disorders are only found in the eye. No other tissue or organ has been documented to be damaged. Presumably this is because of the functional redundancy provided by the existence of another protein, REP2, also known as CHML (choroideremia-like protein), a redundancy which is not complete in the eye [14]. REP1 and REP2 are widely expressed and share 90% similarity and 75% identity in their amino acid composition. In animal models, *Chm* knockout (KO) is surprisingly severe, causing embryonic lethality in males, due to abnormalities in extraembryonic mouse tissues such as the placenta and yolk sac [15]. Carrier females (*Chm*^null/WT^) are viable and exhibit progressive retinal degeneration [12, 16]. To further investigate the pathogenesis of the disease, we generated conditional *Chm* mouse models in which *Chm* is knocked out specifically in retinal progenitor cells (RPCs). Our conditional *Chm* KO strategy avoids embryonic lethality while recapitulating human retinal degeneration timelines, a critical advancement over prior models. Our goal was to determine whether targeted *Chm* KO in RPCs could initiate or affect the degeneration of the retina. We find that REP1 is necessary for normal PR function and synaptic wiring, and plays an important role in phototransduction and visual function. Additionally, we proved that the retinal degeneration in *Chm*-deficient mouse is associated with microglial activation mediated by the NF-κB signaling pathway. This indicates that focusing on neuroinflammation could represent a promising therapeutic approach for patients with choroideremia.

## Materials and methods

### Generation of retinal conditional *Chm* KO animals

To generate a floxed *Chm* allele, we inserted *loxP* sites in introns 4 by homologous recombination. Genotypes were confirmed by PCR from tail genomic DNA samples. Conditional KO mouse were obtained by crossing floxed *Chm* allele mouse with the *Six3-Cre* transgenic line to delete exon 4 of *Chm* in the developing retina to produce the *Chm*^*fl/y*^*, Chm*^*fl/+*^*; Six3-Cre* and *Chm*^*fl/y*^*; Six3-Cre* mouse, and maintained by breeding with C57BL6/J (wild type) mouse. *Chm* alleles using primers *Chm*-loxP-F, CAATTAGTCTAATAAGCA, and *Chm*-loxP-R, TCCTCAAGTTATTCACTACC. *Six3-cre* were genotyped using the generic Cre primers Cre-F, TCGATGCAACGAGTGATGAG, and Cre-R, TTCGGCTATACGTAACAGGG.

All experimental protocols were conducted in accordance with NIH Office of Laboratory Animal Welfare standards and approved by the Wuhan University Laboratory Animal Center. Subjects were housed in specific pathogen-free facilities with controlled atmospheric conditions (22 ± 1°C, 55 ± 5% humidity) under standardized photoperiodic regulation (12 L:12D cycle). After the randomized allocation of animals to the groups, samples are coded and recorded until the data are analyzed.

### Optical coherence tomography (OCT) imaging

Mouse were anesthetized via intraperitoneal injection prior to ocular examinations. Pupillary dilation was achieved through topical administration of 0.5% compound tropicamide ophthalmic solution. Corneal hydration was maintained during procedures through periodic application of physiological saline. Retinal imaging was performed using an OCT system (Beijing HealthOlight Technology, China) coupled with a fundus photography apparatus. Subjects were immobilized on a stereotaxic platform with corneal contact established against the imaging lens. Linear horizontal scanning protocols were implemented to obtain comprehensive retinal cross-sections. OCT parameters were optimized for maximal signal-to-noise ratio through real-time focus adjustment and application of averaging algorithms, with subsequent image acquisition upon achieving satisfactory resolution.

### Hematoxylin and eosin staining (H&E staining)

Following cervical dislocation euthanasia, ocular globes were immediately enucleated and fixed in 4% paraformaldehyde (PFA, 80096618, Sinopharm Chemical Reagent) for 24 h at 4 °C. Sequential tissue processing included dehydration through an ethanol gradient series, xylene-mediated clearing, and paraffin embedding. Serial sections of 5 µm thickness were obtained using a precision microtome (RM2016, Leica Biosystems). Deparaffinized sections underwent standard H&E staining protocol: nuclei were counterstained with Mayer’s hematoxylin (5 min) followed by cytoplasmic eosin Y differentiation (5 min), with intermittent distilled water rinses between steps. Histological evaluation was performed through brightfield microscopy (E100, Nikon), with representative retinal sections digitally captured.

### Immunofluorescence

Following euthanasia via cervical dislocation, ocular globes were immediately enucleated and dissected in ice-cold phosphate-buffered saline (PBS, G0002, Servicebio) to remove anterior segments, lenses, and vitreous bodies. Retinas were isolated and fixed in 4% PFA for 40 min at 4 °C. Tissue processing included sucrose gradient dehydration, embedding in Optimal Cutting Temperature compound (4583, SAKURA), and flash-freezing at –80 °C. Retinal cryosections (14 µm thickness) were prepared using a cryostat (CM1950, Leica Biosystems). Sections were blocked for 1 hour at room temperature with a solution containing 4% bovine serum albumin (BSA, 4240GR100, Biofroxx) and 0.5% Triton X-100 (93289, Sigma-Aldrich). Primary antibodies (listed in Supplementary Table S1) were applied for overnight incubation at 4 °C, followed by species-matched fluorescent secondary antibodies and nuclear counterstaining with DAPI (1 µg/mL). Imaging was performed using a confocal laser-scanning microscope (LSM880, Carl Zeiss) equipped with ZEN imaging software. Quantitative analysis of fluorescence intensity was conducted using ImageJ/Fiji (National Institutes of Health).

### Retinal function test ERG

All experimental procedures were executed under infrared-attenuated illumination following overnight dark adaptation. Mouse were anesthetized via intraperitoneal injection and subjected to pupillary dilation through topical administration of 0.5% compound tropicamide ophthalmic solution. Full-field ERG responses were recorded across standardized flash intensities spanning two photometric ranges: scotopic (0.001–10 cd·s/m^2^) and photopic (0.1–100 cd·s/m^2^), with explicit quantification of a-wave (PR response) and b-wave (bipolar cell, BC activity) amplitudes. Signal acquisition utilized a corneal electrode-coupled RetiMiner 4.0 system (IRC Medical Equipment, Chongqing, China) under controlled background illumination (30 cd/m^2^ for photopic adaptation). Raw waveforms were analyzed using manufacturer-specific protocols, with statistical comparisons performed in GraphPad Prism 7 (GraphPad Software, La Jolla, CA).

### RNA-sequencing (RNA-Seq) analysis

Following RNA extraction from P14 retinas by TRIzol Reagent (Invitrogen, 10296028), DNA digestion was performed by DNaseI. The quality and integrity of RNA were assessed by A260/A280 ratio (Thermo Fisher, USA) and agarose gel electrophoresis, respectively. The stranded RNA sequencing library was prepared with 2 g total RNAs following the manufacturer’s instruction for Illumina® (Seqhealth Co., Ltd. DR08402). 200–500 bps PCR products were enriched, quantified, and sequenced with the PE150 model (Illumina, USA). Raw sequencing data were first filtered by Trimmomatic v0.36. Using STRA software with default parameters, clean data were categorized to the retina reference genome. RPKMs were determined after featureCounts counted reads corresponding to each gene’s exon regions. The edgeR program v 3.12.1 was used to acquire genes that were differentially expressed between groups. Statistical analysis of differentially expressed genes was formed by Gene ontology analysis and Kyoto encyclopedia of genes and genomes enrichment analysis (KOBAS v2.1.1). A *p* value cutoff of 0.05 and a fold-change cutoff of 2 was used to calculate the statistical differences in gene expression.

### Real-time quantitative reverse transcription-PCR (RT-PCR)

Retinal RNA isolation was performed in quadruplicate biological replicates (two retinas per experimental group) using TRIzol Reagent (10296028, Invitrogen) following mechanical homogenization. Fluorescence quantitative PCR with SYBR Green I fluorescent dye (Q111-02/03, Vazyme Biotech) was used to measure gene transcription levels in accordance with the manufacturer’s recommendations. The results were standardized to endogenous gapdh as an internal control. Primers utilized for RT-PCR are listed in Supplementary Table S2.

### Transmission electron microscopy (TEM)

Retinal ultrastructural analysis was performed on postnatal day 30 mouse euthanized via isoflurane overdose with cervical dislocation confirmation. The eyes were injected with 2.5% glutaraldehyde (abs9277, Absin), and 1% PFA, then immersion was fixed overnight in the same fixative. Tissue processing included graded methanol dehydration, osmium tetroxide post-fixation, and epoxy resin embedding. Retinal tissue was subsequently serially sectioned at 70- to 90-nm onto polyvinyl formal resin–coated gold slot grids. Each section was imaged by automated TEM at 2.18 nm resolution with more than 1000 image tiles per section stored in 16- and 8-bit versions, creating image pyramids for web visualization of a 0.25-mm diameter volume of the mouse retina, viewed and annotated with the Viking viewer.

### Western blot

To extract total proteins, RIPA Buffer was mixed with protease inhibitor cocktails and phosphatase inhibitors (Sigma-Aldrich), the samples were transferred to polyvinylidene fluoride (PVDF) membranes (Millipore) after SDS-PAGE was performed. These membranes were first blocked in 5% skim milk at room temperature, then incubated with primary antibodies at 4 °C overnight (antibody details are listed in Supplementary Table S1). After this, the membranes were incubated with horseradish peroxidase-conjugated secondary antibodies at room temperature for 2 h. Chemiluminescence signals were detected via SuperPico ECL Chemiluminescence Kit (Vazyme), and ImageJ (V. 1.8.0) software was utilized for signal visualization and analysis.

### Statistical analysis

Results were displayed as the mean ± SEM using Prism 7.0 (GraphPad, USA) for quantitative analysis. Data were examined using the Student’s *t* test (SPSS 15.0.1, USA). Differences were assessed as statistically significant at *P* < 0.05.

## Results

### Inactivation of *Chm* induces retinal neurons impairment

**Fig. 1:**
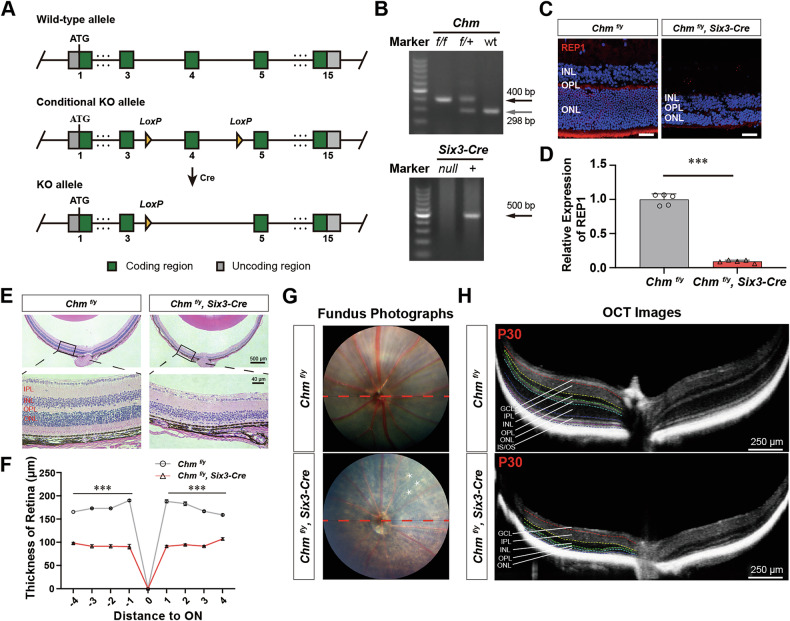
Inactivation of *Chm* induces retinal neuron impairment. **A**, **B** *Floxed Chm* mouse was crossed with *Six3-Cre* transgenic mouse to conditionally ablate *Chm* in the retina. Genotypes (*Chm*^*fl/y*^*, Chm*^*fl/y*^*; Six3-Cre*) were determined by PCR. **C** Immunostaining of retinal sections labeled with antibodies against REP1 (red) and DAPI (blue) from *Chm*^*fl/y*^*; Six3-Cre* mouse and littermate controls. Scale bar, 30 µm. **D** The relative expression of REP1 protein in the retinas of *Chm*^*fl/y*^ and *Chm*^*fl/y*^*; Six3-Cre* mouse observed by immunofluorescence staining (*n* = 5). **E**, **F** Representative magnified H&E staining at P30 between *Chm*^*fl/y*^*; Six3-Cre* mouse and littermate controls (*n* = 5). **G**, **H** Fundus photography and optical coherence tomography (OCT) to observe fundus changes in mouse after *Chm* KO. ONL outer nuclear layer, OPL outer plexiform layer, INL inner nuclear layer, IPL inner plexiform layer GCL ganglion cell layer, IS/OS inner segments/ outer segments.

*Chm*^*fl/fl*^ mouse were crossed with the *Six3-Cre* transgenic line to produce *Six3-Cre* (*Chm*^*fl/y*^*; Six3-Cre*) mouse (Fig. 1A), which results in deletion of *Chm* in the retina and ventral forebrain. The mouse genotypes (*Chm*^*fl/y*^*; Six3-Cre*) were determined by PCR (Fig. 1B). Immunofluorescence labeling confirmed that expression of REP1 protein in the retina was drastically reduced in *Chm*^*fl/y*^*; Six3-Cre* mouse compared with control heterozygous *Chm*^*fl/y*^ mouse (Fig. 1C, D). H&E staining showed rapid and progressive degeneration of PR cells (Fig. 1E). At postnatal day 30 (P30), the retinal thickness of *Chm*^*fl/y*^ mouse was 188.1 ± 3.457 μm. Retinal thickness in *Chm*^*fl/y*^*; Six3-Cre* mouse was reduced by nearly half (91.2 ± 2.341 μm) (Fig. 1F). Changes in thickness of the IS/OS and ONL layers were the most apparent. We examined retinal structure in vivo. The retinal fundus of mouse in the control *Chm*^*fl/y*^ group had normal retinal structure, and the optic papilla was located in the center of the posterior pole of the eye. Large radial blood vessels emanating from the optic papilla can be seen. The diameter of the blood vessels was normal, and blood flow in the lumen of the vessels was clearly visible. Compared to control mouse, *Chm*^*fl/y*^*; Six3-Cre* mouse exhibited pigmented retina, a narrow fundus vascular diameter, and pale papilla (Fig. 1G). OCT scans showed that the thickness of each layer of the retina was reduced, to a similar extent as the results of HE staining (Fig. 1H). These results suggest that *Chm* deficiency causes dramatic retinal abnormalities.

### Ablation of *Chm* resulted in decreased number of retinal neuron cells

**Fig. 2:**
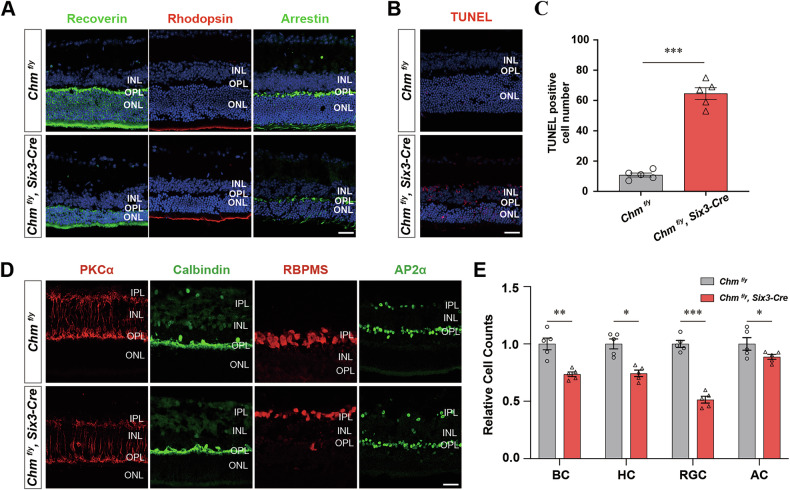
Ablation of *Chm* resulted in apoptosis and decreased numbers of retinal neurons. **A** Retinal cross-sections of *Chm*^*fl/y*^ and *Chm*^*fl/y*^*; Six3-Cre* mouse, stained for TUNEL (red) and DAPI (blue). **B** Quantitation of TUNEL+ cells in P30 *Chm*^*fl/y*^ and *Chm*^*fl/y*^*; Six3-Cre* mouse retinas. Results are presented as the mean ± SEM (*n* = 5). **C** Quantitation of TUNEL positive cells in P30 *Chm*^*fl/y*^ and *Chm*^*fl/y*^*; Six3-Cre* mouse retinas (*n* = 5). **D** Neuronal marker was used to recognize nerve distributions in retina layers, indicating the neuronal injury in *Chm*^*fl/y*^*; Six3-Cre* mouse. Scale bar: 30 μm. **E** Quantitation of cells that were immunoreactive for Neuronal marker in P30 *Chm*^*fl/y*^ and *Chm*^*fl/y*^*; Six3-Cre* mouse retinas (*n* = 5). *P < 0.05, **P < 0.01, ***P < 0.001.

PR damage is an important indicator of disease progression in CHM patients. We performed immunofluorescent labeling using antibodies against several cell type-specific markers including Recoverin (PRs), Rhodopsin (rods), Arrestin (cones). Immunofluorescence labeling showed decreased numbers of PRs, in agreement with morphological measurements of retinal layers thinning at P30. PRs immunolabeled with anti-recoverin antibodies were reduced to 5–7 cell rows, compared to littermate controls (13-16 cell rows), and the OS layer was disorganized. Cones labeled by anti-arrestin antibodies had lightly stained somas, and the axons were not visible. Rods immunolabeled by anti-rhodopsin antibodies showed disrupted structural integrity, and sparse pedicles (Fig. 2A). TUNEL staining showed significant apoptosis of *Chm*^*fl/y*^*; Six3-Cre* mouse associated with retinal degeneration. The number of TUNEL-positive cells in *Chm*^*fl/y*^*; Six3-Cre* retina were significantly increased compared with control mouse (Fig. 2B, C). TUNEL-positive cells were also expressed in the outer plexiform layer (OPL), inner nuclear layer (INL), and ganglion cell layer, suggesting that the apoptosis was not limited to PR cells. We next performed immunofluorescent labeling using antibodies against several cell type-specific markers including PKCα (rod BCs), Calbindin (horizontal cells, HCs), RNA binding protein (retinal ganglion cells), and adapter protein2α (amacrine cells, ACs). Anti-PKCα antibodies revealed a decrease in both length and density of dendritic processes of rod BCs in the OPL of *Chm*^*fl/y*^*; Six3-Cre* mouse. Anti-calbindin antibody-labeled HC somas and punctate-stained dendrites showed irregular protrusions and loss of dense plexus of processes. The number of labeled ganglion cells, which ramify in multiple layers of the IPL, was significantly decreased in *Chm*^*fl/y*^*; Six3-Cre* mouse. There was no significant difference in either the morphology or number of AP2α-labeled amacrine cells (Fig. 2D, E).

### Ablation of *Chm* in retina significantly attenuated visual function

**Fig. 3:**
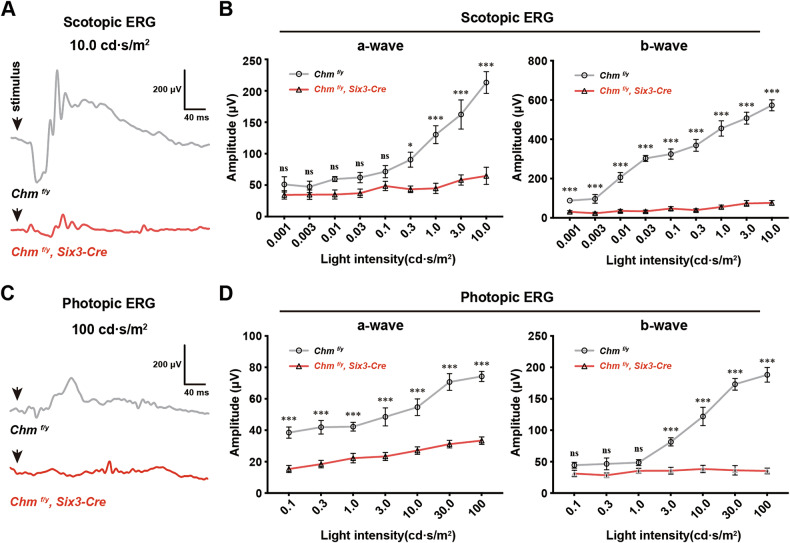
Ablation of *Chm* in the retina significantly attenuated visual function. **A** Representative ERG traces elicited by scotopic conditions at a flash intensity of 0.1 cd•s/m^2^. **B** The plot of scotopic ERG a-wave and b-wave amplitudes elicited from *Chm*^*fl/y*^*; Six3-Cre* mouse and littermate controls were recorded at P30. (mean ± SEM, n = 10; *: *P* < 0.05, **: *P* < 0.01, ***: *P* < 0.001, ns: no significance). **C** Representative ERG traces elicited by photopic conditions at a flash intensity of 100 cd•s/m^2^. **D** The plot of photopic ERG a-wave and b-wave amplitudes elicited from *Chm*^*fl/y*^*; Six3-Cre* mouse and littermate controls were recorded at P30. (mean ± SEM, *n* = 10; ***: *P* < 0.001, ns: no significance).

We next measured ERGs to assess the impact of *Chm* inactivation on retinal function at P30. In the *Chm*^*fl/y*^*; Six3-Cre* cohort, the scotopic a-wave of the combined rod-cone response was reduced by 69.6% compared to the control group (64.78 ± 13.63 μV versus 213.26 ± 17.34 μV, *P* < 0.001), and the scotopic b-wave, which reflects response from rod BCs, was reduced by 86.7% at a light intensity of 10 cd·s/m^2^ (76.19 ± 12.37 μV versus 573.03 ± 27.96 μV, *P* < 0.001) (Fig. 3A, B). The photopic ERG reflects response from cone BCs, the a-wave response was reduced by 55% compared to that of the control (33.43 ± 2.39 μV versus 74.26 ± 3.22 μV, *P* < 0.001), and the photopic b-wave was reduced by 81.3% compared with the control at an intensity of 100 cd·s/m^2^ (35.24 ± 4.57 μV versus 188.14 ± 11.67 μV, *P* < 0.001). At most light intensities, the scotopic and photopic ERG a/b wave amplitudes of *Chm*^*fl/y*^*; Six3-Cre* mouse exhibited a significant decrease (Fig. 3C, D). In summary, our results demonstrated that *Chm* deficiency leads to dramatic visual dysfunction.

### REP1 is required for the development of PRs, cation channel activity, synapse formation and phototransduction

**Fig. 4:**
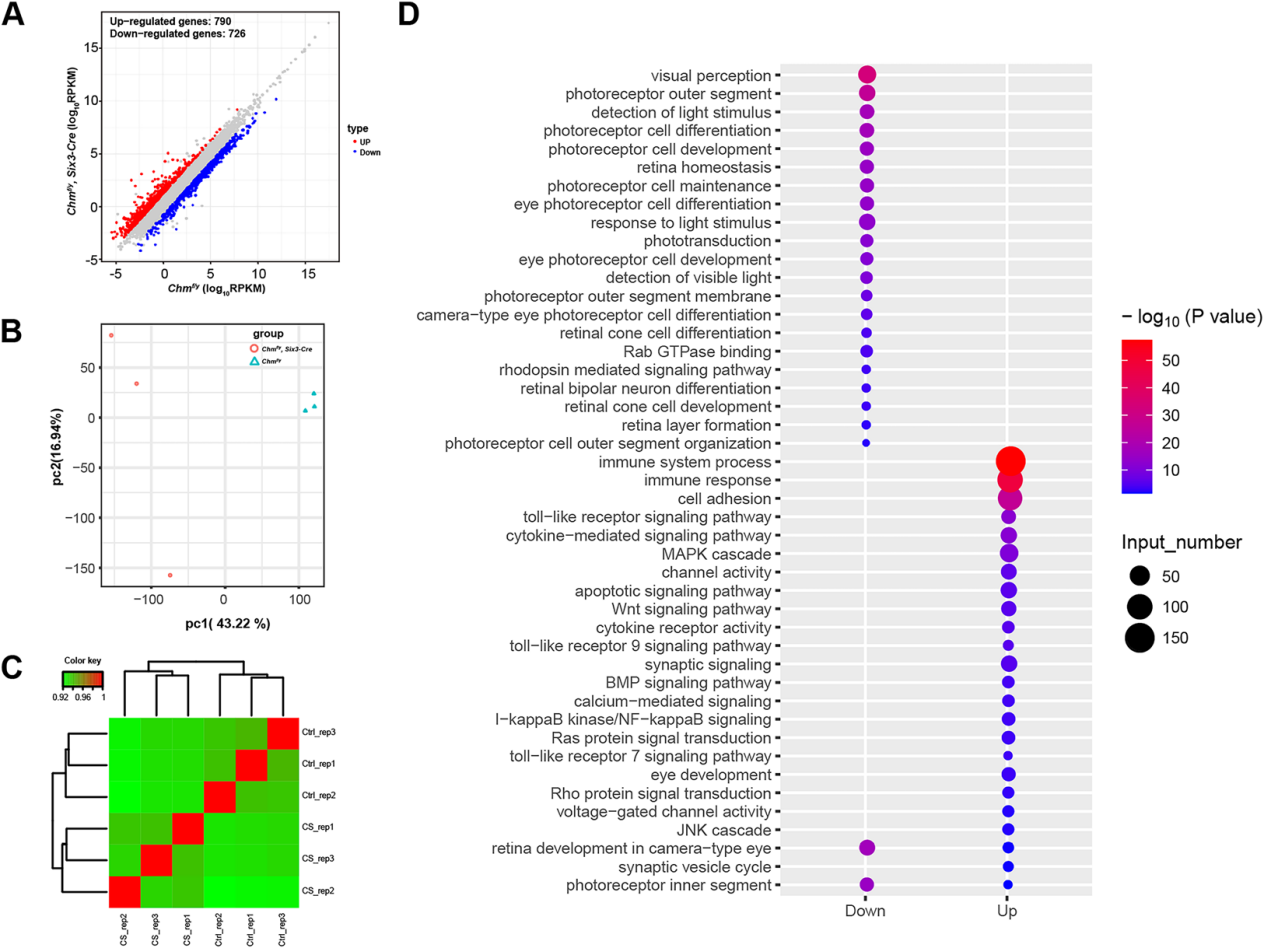
Up- and down–regulation of select gene clusters in *Chm*^*fl/y*^*; Six3-Cre* mouse. **A**–**C** Scatter plots and heat maps of differential gene expression profiles were compared by Spearman correlation coefficient (*n* = 3 for each group). A total of 1516 transcripts exhibited differential expression (downregulation, blue; upregulation, red) between *Chm*^*fl/y*^*; Six3-Cre* mouse, and littermate controls. **D** GO analysis evidenced decreases in gene clusters related to visual perception, detection of light stimulus, retina homeostasis, and phototransduction. Increases in gene clusters related to toll-like receptor signaling pathway, MAPK cascade, channel activity, Wnt signaling pathway, synaptic signaling, BMP signaling pathway, I-kappaB kinase/NF-kappaB signaling, and Ras protein signal transduction.

Using RNA-seq, we examined differential expression of genes in three biological replicates, comparing retinas of P30 *Chm*^*fl/y*^*; Six3-Cre* mouse with control littermates. We found 790 upregulated and 726 downregulated genes in *Chm*^*fl/y*^*; Six3-Cre* mouse compared to littermate controls (Fig. 4A–C). We used gene ontology (GO) analysis to objectively identify up and downregulated gene categories in *Chm*^*fl/y*^*; Six3-Cre* mouse. GO analysis revealed overall decreases in gene clusters related to visual perception, detection of light stimuli, retina homeostasis and phototransduction. Conversely, there were increases in gene clusters related to the toll-like receptor signaling pathway, MAPK cascade, channel activity, Wnt signaling pathway, synaptic signaling, BMP signaling pathway, I-kappaB kinase/NF-kappaB signaling, and the Ras protein signal transduction (Fig. 4D). Altogether, the RNA-seq data reveal a pattern in which neural development and growth-related transcriptomes are the most downregulated pathways in the *Chm*^*fl/y*^*; Six3-Cre*, followed by, phototransduction and synaptic pathways.

### REP1 deficiency leads to a decrease of retinal cell and synapse genes

**Fig. 5:**
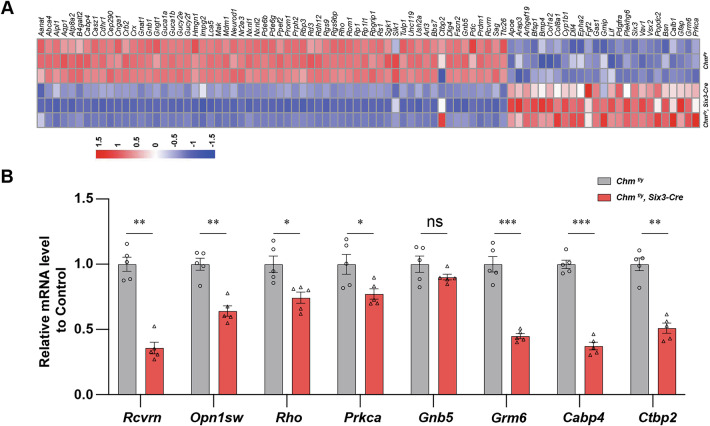
REP1 deficiency decreases key PR and ON BC mRNA transcripts. **A** The expression levels of PR transcription factors from retinal extracts between *Chm*^*fl/y*^*; Six3-Cre* mouse and littermate controls are represented as a heat map. **B** Transcript-level differential expression of PR (*Rcvrn, Opn1sw, Rho*), BC (*Prkca*), synapse (*Grm6, Cabp4, Ctbp2*) related genes was confirmed by RT-PCR between *Chm*^*fl/y*^*; Six3-Cre* mouse and littermate controls at P14 (mean ± SEM, *n* = 5; *: *P* < 0.05; **: *P* < 0.01; ***: *P* < 0.001, ns: no significance).

RNA-seq results indicate that levels of several PR OS/inner segment (IS) molecules were impacted by the absence of REP1 (Fig. 5A). RT-PCR analyses reveals the expression of PR (*Rcvrn, Opn1sw, Rho*), BC (*Prkca*), synapse (*Grm6, Cabp4, Ctbp2*) related genes was decreased at P30 in *Chm*^*fl/y*^*; Six3-Cre* mouse compared with littermate controls (Fig. 5B). This is consistent with the decrease in the PR a-wave as well as thinning of the PR layer.

### REP1 deficiency affected synapse formation and phototransduction

**Fig. 6:**
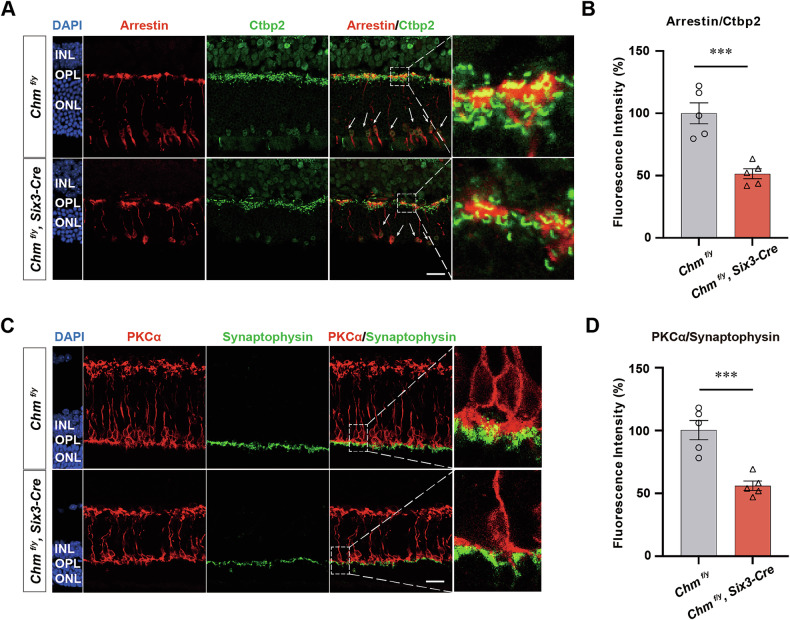
REP1 deficiency affected synapse formation and phototransduction. **A** Presynaptic ribbons Ctbp2 (green) co-immunostained with Arrestin (red) in retinal sections. Arrows indicate the cell bodies and dendritic tips of RBCs. **B** Quantitation of cells that were immunoreactive for Ctbp2 marker in P30 *Chm*^*fl/y*^ and *Chm*^*fl/y*^*; Six3-Cre* mouse retinas (*n* = 5). ***P < 0.001. **C** Presynaptic ribbons synaptophysin (green) were co-immunostained with antibodies against RBC-specific marker PKCα (red) at P30 retinal sections. **D** Quantitation of cells that were immunoreactive for synaptophysin marker in P30 *Chm*^*fl/y*^ and *Chm*^*fl/y*^*; Six3-Cre* mouse retinas (*n* = 5). ***P < 0.001. Scale bar, 30 µm.

Significant disruptions were observed in the structural organization of cone photoreceptors. Specifically, the characteristic morphology of cones was markedly altered, accompanied by a loss of their regular parallel alignment within the retinal layers (Fig. 6A). RT-PCR analyses reveals a downregulation of transcripts for the postsynaptic gene *Grm6* and for the presynaptic ribbon protein *Ctbp2*, both of which are essential for synaptic transmission between PRs and ON BCs. Immunofluorescence labeling with anti-PKCα antibody indicate a decreased number of ON BCs, accompanied by axonal failure at P30 in *Chm*^*fl/y*^*; Six3-Cre* mouse. To explore the causes of the diminished scotopic b-wave, ON BCs were colabeled with antibodies against PKCα antibody (red), and PRs were labeled with an antibody to the presynaptic ribbon protein synaptophysin (green) at P30 retinal sections (Fig. 6C). The number of Ctbp2 and synaptophysin, which ramify in multiple layers of the IPL, were significantly decreased by 48.9% and 52.1% respectively compared to that of the control (Fig. 6B, D).

### REP1 deficiency affected ribbon and HCs

**Fig. 7:**
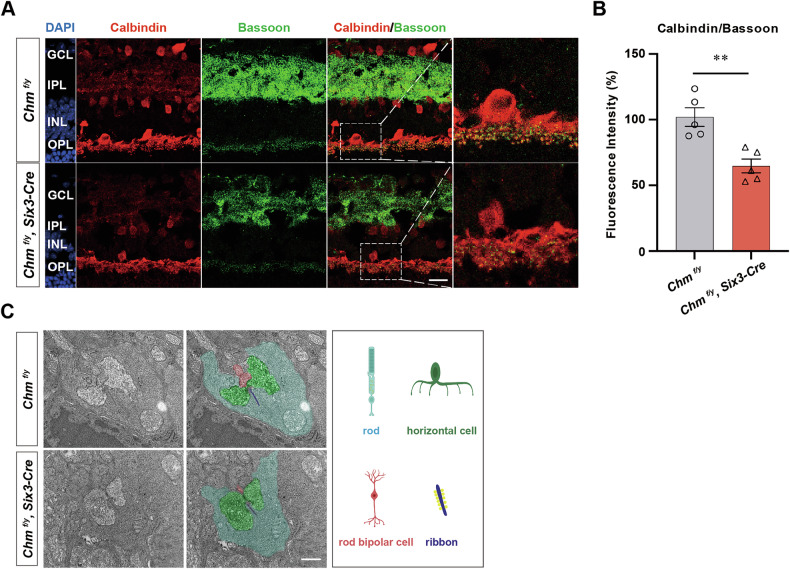
Altered synaptic triad structure after knockdown of the *Chm* gene. **A** Frozen sections and immunofluorescence staining maps of retinas from P30-day *Chm*^*fl/y*^*; Six3-Cre* and *Chm*^*fl/y*^ mouse with HC-specific antibody Calbindin (red) and presynaptic marker Bassoon (green) (*n* = 5). Scale bar: 50 μm. **B** Quantitation of cells that were immunoreactive for Bassoon marker in P30 *Chm*^*fl/y*^ and *Chm*^*fl/y*^*; Six3-Cre* mouse retinas (*n* = 5). **P < 0.01. **C** The typical ribbon synapse is an arch-shaped electronically highly dense structure located at the angle of the triplet structure. Mouse retinal ribbon synapses are flanked by vesicular structures. It is the site of invagination formed by adjacent HCs. The downstream BC dendrites are also involved in forming the triad structure. A few residual ribbon synapses observed in *Chm*^*fl/y*^*; Six3-Cre* group mouse with shortened lengths, blurred structural boundaries, and lacking the typical invaginated synaptic structures. Scale bar: 400 nm.

In normal retina, HC and ON BC dendrites form synaptic connections with both rods and cones. Thus, both rods and cones are contacted by HCs and either rod or cone type ON BCs, forming a classical triad in which a single BC occupies the central position and HC dendrites occupy the lateral positions flanking the BC dendrite. At the sites of contact, Bassoon is expressed at presynaptic terminals of PRs. Compared with the control group, the *Chm*^*fl/y*^*; Six3-Cre* group mouse showed reduced Bassoon immunofluorescence at sites of synaptic contact, and loss of the typical horseshoe-shaped morphology, characterized by an irregular shape and position of the rod BC apical dendrites. Furthermore, antibody staining of the HC marker Calbindin (green) and Bassoon (red), revealed that Bassoon and Calbindin co-labeled significantly less and were discontinuously distributed in the retina. Furthermore, punctate Calbindin labeling that is normally found in HC processes of control mouse was sparse in the *Chm*^*fl/y*^*; Six3-Cre* mouse line, the dendrites of HCs were flattened and narrowed, and the number of synaptic junctions between PRs and HCs was reduced (Fig. 7A). The number of Bassoon dots in OPL were decreased by 61.1% compared to that of the control (Fig. 7B).

We also combined high-resolution TEM to probe for changes in synaptic ultrastructural after knockout of the *Chm* gene. The typical ribbon synapse is an arch-shaped electronically highly dense structure located at the angle of the triad structure. Vesicles are tethered to synaptic ribbons bilaterally. The structural integrity of the ribbon and associated vesicles ensures the rapid and continuous release of transmitter from PR terminals (Fig. 7C). Postsynaptically, the classic triad comprised of the invaginating ON BC dendrite and flanking HC processes is evident. In the *Chm*^*fl/y*^*; Six3-Cre* mouse line, only a few residual ribbon synapses characterized by shortened lengths and blurred structural boundaries could be observed. Furthermore, postsynaptic sites lacked the typical invaginating synaptic structure.

### REP1 deficiency leads to microglial and Müller activation

**Fig. 8:**
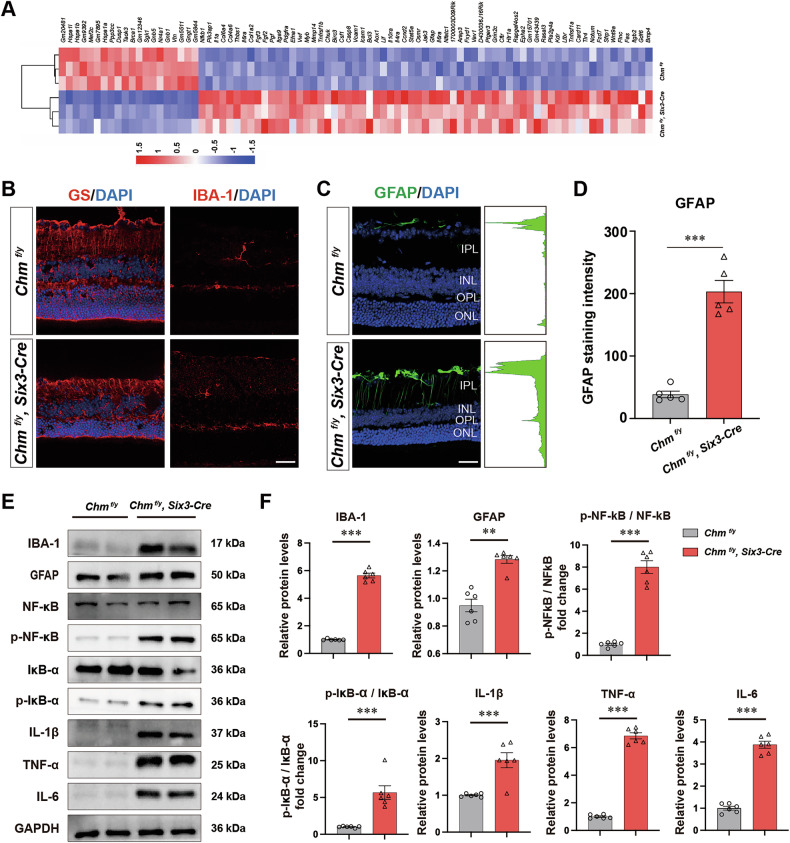
REP1 deficiency leads to Microglial and Müller activation. **A** The expression levels of pro-inflammatory cytokines and chemokines from retinal extracts between *Chm*^*fl/y*^*; Six3-Cre* mouse and littermate controls are represented as a heat map. **B**, **C** Retinal sections immunostained with antibodies against IBA-1 (green) to identify the morphology of microglial cells and GS and IBA-1 to detect reactive Müller cells. Representative images of GFAP immunostaining on retinal sections. **D** Quantitation of cells that were immunoreactive for GFAP in P30 *Chm*^*fl/y*^ and *Chm*^*fl/y*^*; Six3-Cre* mouse retinas (*n* = 5). ONL outer nuclear layer, OPL outer plexiform layer, INL inner nuclear layer, IPL inner plexiform layer. Scale bar: 50 µm. **E**, **F** Western blot of protein showed increased expression levels of IBA-1, GFAP, NF-κB, p- NF-κB, p-IκB-α, TNFα, IL-1β and IL-6 in the *Chm*^*fl/y*^ and *Chm*^*fl/y*^*; Six3-Cre* mouse retinas (*n* = 6). **: *P* < 0.01, ***: *P* < 0.001.

RNA sequencing revealed an upregulation of glial and immune‑response genes in the *Chm*^*fl/y*^*; Six3-Cre* mouse retina (Fig. 8A). We evaluated the activity of GS and IBA-1+ cell populations in *Chm*^*fl/y*^*; Six3-Cre* mouse, GS and IBA-1+ cells were shown to be more prevalent in the *Chm*^*fl/y*^*; Six3-Cre* mouse (Fig. 8B). Additionally, these cells demonstrated migratory behavior towards the photoreceptor layers, specifically targeting the ONL and OSs, indicating microglial and Müller cells activation in this *Chm*^*fl/y*^*; Six3-Cre* retina. The GFAP immunoreactivity of was significantly increased, which indicate the reactive gliosis of astrocytes cells. Immunofluorescence indicates an increased count of activated microglia (identified by swollen bodies and short processes) was observed in the *Chm*^*fl/y*^*; Six3-Cre* retina (Fig. 8C, D), as compared with the resting ones with highly ramified processes in the control.

In addition, western blot results of IBA-1, GFAP further verified microglia were activated. And the up-regulated phosphorylation of NF-κB and IκB-α suggested that the change of retinal microglia is related to activation of the NF-κB pathway. We further verified the expression of the NF-κB pathway relevant molecules, the expression levels of proinflammatory markers, including TNF-α, IL-1β, and IL-6, were upregulated in *Chm*^*fl/y*^*; Six3-Cre* mouse (Fig. 8E, F). Those results suggest that NF-κB pathway likely contributes to the degenerated progress of CHM mouse model.

## Discussion

PRs are responsible for capturing light, while BCs transmit light information to the inner retina [17]. The ERG is a useful tool to assess function of both PR (a-wave) and ON BCs (b-wave) [18]. In the present study, we found that elimination of *Chm* expression almost completely extinguished both the a-wave and b-wave, consistent with a high degree of damage and dysfunction of both PRs and BCs (Fig. 3A–D). The decrease in the scotopic a-wave is most likely due to a loss of transduction machinery in addition to reduced PR numbers. The loss of the b-wave is caused by the rod photoreceptors death and synaptic abnormalities. Meanwhile, our study suggests that the target cells for treatment are not only limited to photoreceptors, and that the repair of synaptic connections is also of great value for the recovery of visual function in CHM patients. Fundus photography reveals central and midperipheral mottling; intravenous fluorescein angiography shows areas of RPE atrophy; OCT shows outer retinal loss correlating with the areas of RPE degeneration.

*Chm* ablation leads to retinal degeneration, indicating that REP1 is a critical regulator of retinal homeostasis and survival. We attempted to explore the mechanism of retinal hypoplasia and decreased visual function in *Chm*-deficient mouse. Using RNA-seq to measure gene expression, followed by GO analysis to interpret the results, we found that genes associated with the inner and outer segments of PRs were expressed at significantly lower levels compared to the control group. Our study supports the idea that REP1 is required for cone survival and regulates localization of cone cyclic nucleotide-gated channels to the OS. The morphology of the cones was disrupted, and their regular parallel alignment was lost (Fig. 6A). Rods and cones in the OPL form selective synaptic connections with BC and HC terminals [19, 20]. Our further analysis indicates that ablation of *Chm* in the retina interfered with BCs structure and function. Somas of BCs lack the highly organized packing and their axons have shrunk in length (Fig. 6B). Additionally, RNA-Seq indicates that elimination of *Chm* affected the expression levels of key molecules involved in synapse and phototransduction molecules (Fig. 5A).

The presynaptic ribbon is a highly organized structure in the PR terminal near the region of the membrane vesicles. Ctbp2, also known as RIBEYE, is a key component of the synaptic ribbon in both rods and cones. Decreased numbers of Ctbp2 puncta were detected in *Chm*^*fl/y*^*; Six3-Cre* mouse, indicating either a reduced number of synapses, or Ctbp2-expressing ribbons (Fig. 6A). However, the concomitant decrease in Bassoon expression makes the former possibility more likely. Thus, as stated above, the loss of the b-wave is likely due to a combination of pre- and postsynaptic abnormalities. HCs make synaptic connections with postsynaptic dendrites and modulate synaptic transmission between PRs and BCs [21, 22]. The number of synaptic terminals and the distributed regularity in BCs and HCs is determined by the strength of synaptic input from presynaptic cones [23, 24]. HCs that were positively labeled by Calbindin also revealed degenerative alterations in *Chm*^*fl/y*^*; Six3-Cre* mouse, followed by a progressive loss of PR synaptic ribbons co- immunostained by Bassoon (Fig. 7A).

Microglia are specialized immune cells in the brain and retina, are typically maintained in a quiescent state under physiological conditions. Activated microglia have been implicated in the progress of neurodegenerative disorders, and the existence of such activated cells correlates with increased impairment to neurons [25]. However, under pathological circumstances that are chronic or severe, the continuous activation of microglia might lead to the increase of pro-inflammatory cytokines and chemokines [26]. The inflammatory status of the retina was evaluated in our study by examining GS and IBA-1 positive cells, and we observed phenotypic alterations in cell populations associated with immune and inflammatory reactions. GFAP positive cells indicate that microglial cell activation occurs as neuroinflammation advances, and this is accompanied by the activation of astrocytes and Müller cells, these events contribute to a further enhancement of the inflammatory response. NF-κB regulates the activity of numerous target genes involved in immune responses and inflammatory processes. The phosphorylate IκB leads to degradation and release of NFκB, then subjected to additional post-translational modifications, acetylation included, these modifications facilitate the protein’s translocation to the nucleus and its stable binding to DNA. These coordinated processes are essential for sustaining NF-κB activation and propagating the inflammatory cascade [27]. In the *Chm* KO retina, key components of the necroptotic machinery, including *Nfkb1*, *Tnfrsf1a*, and *Il1a*, were highly expressed. In the retina, neuroinflammation presents with the activation of glial cells (mainly microglia) and the secretion of pro-inflammatory factors, these factors include cytokines such as tumor necrosis factor-α (TNF-α), IL-1β, IL-6, along with signaling proteins NF-κB and IκB-α. Our data reveal that microglial necroptosis holds key importance in *Chm* deficiency-associated retinal neuroinflammation.

**Fig. 9:**
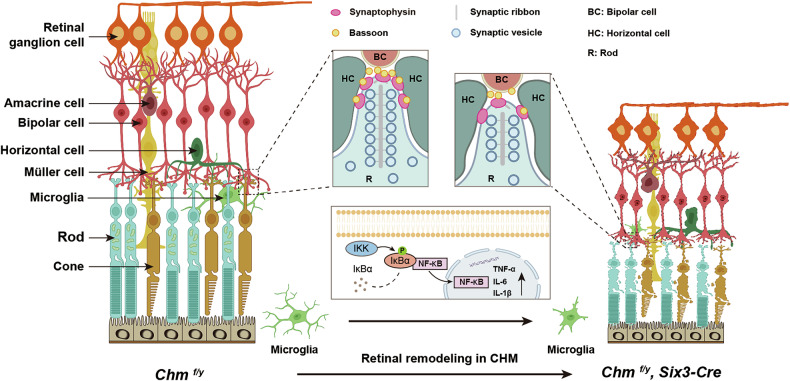
A schematic diagram of REP1 deficiency leads to retinal degeneration. This diagram details the *Chm*-deficiency initiate synapse loss that culminates in functional vision loss, and microglial necroptosis activated by NF-κB pathway, leading to immune response gene activation in the retina (Created by BioRender.com).

In summary, our present work demonstrates that REP1 regulates PRs and synapse formation in mouse and plays an important role in phototransduction and visual function. Furthermore, we speculate that retinal degeneration in *Chm*-deficient mouse is associated with the activation of microglia mediated by the NF-κB pathway (Fig. 9), suggesting that targeting neuroinflammation is one of the potential therapies for choroideremia patients.

## Supplementary information

Supplementary Tables

Original Western blots_CDDIS-25-1664R

## Data Availability

RNA‑seq raw data and processed count matrices have been deposited in GEO under accession GSE308042.
