# Supplementary material for: Deficiency disrupts photoreceptor viability and synaptic integrity in a choroideremia mouse model
Source: Cell Death Dis. 2025 Dec 22;16(1):914. doi: 10.1038/s41419-025-08336-y (PMC12749510; doi:10.1038/s41419-025-08336-y)
Supplement: Supplementary file 1 — Supplementary Tables [file 41419_2025_8336_MOESM1_ESM.docx]

**Supplementary Tables：**

**Table S1.** Primary antibodies used in our study are listed below.

| **Antibody** | **Host** | **Working dilution** | **Company** | **Cat #** |
| --- | --- | --- | --- | --- |
| REP1 | rabbit | 1:1000 | SIGMA | HPA003231 |
| Recoverin | rabbit | 1:1000 | Millipore | AB5585 |
| Arrestin | rabbit | 1:500 | Millipore | AB15282 |
| Rhodopsin | mouse | 1:500 | Millipore | MAB5356 |
| PKCα | goat | 1:500 | Santa Cruz | SC-208-G |
| mGluR6 | rabbit | 1:100 | SIGMA | SAB4501324 |
| CtBP2 | mouse | 1:100 | BD | 612044 |
| Synaptophysin | rabbit |  | CST | 36406S |
| Trpm1 | rabbit | 1:100 | SIGMA | SAB4301014 |
| Calbindin | rabbit | 1：5000 | SWANT | cb38 |
| Bassoon | mouse | 1:1000 | Enzo Life Science | SAP7F407 |
| GFAP | rabbit | 1:200 (IF)  1:500 (WB) | SIGMA | G4546 |
| GS | mouse | 1：5000 | Millipore | mab302 |
| IBA-1 | rabbit | 1：200 | Wako | 019-19741 |
| IBA-1 | rabbit | 1:2000 (WB) | Proteintech | 10904 |
| NF-κB | rabbit | 1:1000 (WB) | CST | 8242T |
| p-NF-κB | rabbit | 1:1000 (WB) | CST | 3033 |
| IκB-𝛼 | rabbit | 1:2000 (WB) | Abcam | AB32518 |
| p-IκB-α | rabbit | 1:2000 (WB) | Abcam | AB92700 |
| TNFα | rabbit | 1:2000 (WB) | Abcam | AB66579 |
| IL-1β | rabbit | 1:1000 (WB) | Proteintech | 16806 |
| IL-6 | rabbit | 1:1000 (WB) | CST | 12912 |

**Table S2.** The primer sequences utilized in this investigation are listed below. Primer sequences were displayed 5’ to 3’.

| **Gene** | **Forward primer** | **Reverse primer** |
| --- | --- | --- |
| Rcvrn | ACGACGTAGACGGCAATGG | CCGCTTTTCTGGGGTGTTTT |
| Opn1sw | CAGCCTTCATGGGATTTGTCT | GTCGCAACTTTTTGTAATGCAGT |
| Rho | CCCTTCTCCAACGTCACAGG | GTAGAGCGTGAGGAAGTTGATG |
| Prkca | GTTTACCCGGCCAACGACT | TCTTTCACCTCATGCACGTTC |
| *Gnb5* | ATGTGCGATCAGACCTTCCTG | GGAGCAGTAGTTGAGTTGTTGAG |
| Grm6 | GCAGAAACATCTGGTTTGCTG | CCTCCTGTTCATAGGTGGAGTC |
| Cabp4 | CTCCCAGAAGATCCCTAAAGGA | CCCTGGCTAGAGGACTGGTC |
| Ctbp2 | GGCAGCGATTGGACAGGATTT | AGGATGGGCATCTCCACAGT |
