# Supplementary figures and images for: Deficiency disrupts photoreceptor viability and synaptic integrity in a choroideremia mouse model
Source: Cell Death Dis. 2025 Dec 22;16(1):914. doi: 10.1038/s41419-025-08336-y (PMC12749510; doi:10.1038/s41419-025-08336-y)

The original western blots of Figure 8E

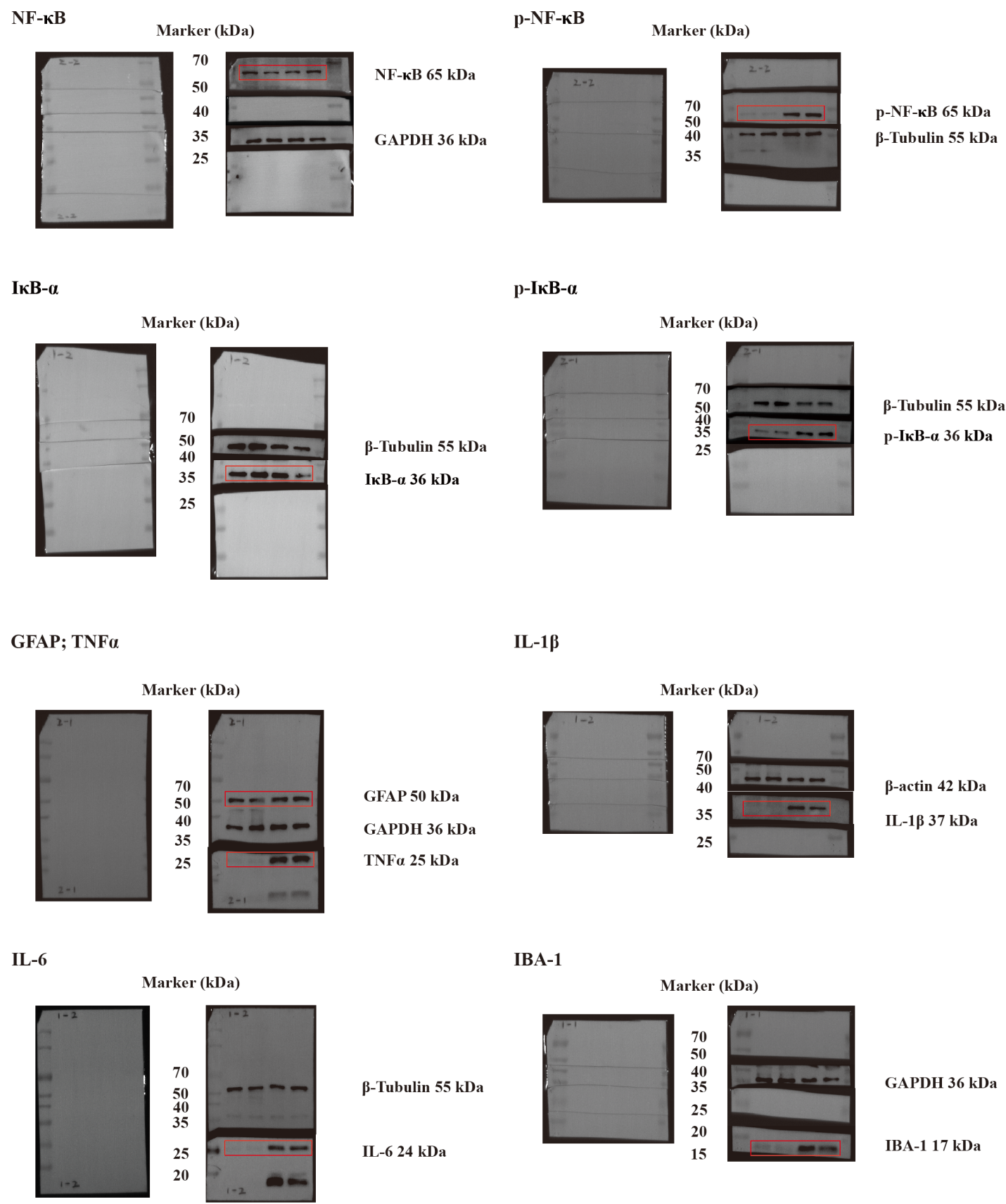

Supplement: Supplementary file 2 — Original Western blots_CDDIS-25-1664R [file 41419_2025_8336_MOESM2_ESM.pdf]
